# Utilization of ring-shaped bone allograft for surgical treatment of adolescent post-tubercular kyphosis: A retrospective study

**DOI:** 10.1097/MD.0000000000007132

**Published:** 2017-06-16

**Authors:** Xiang Yin, Peng Liu, Yao-yao Liu, Wei-Li Fan, Bai-yi Liu, Jian-Hua Zhao

**Affiliations:** Department of Spinal Surgery, Institute of Surgery Research, Daping Hospital, Third Military Medical University, Chongqing, China.

**Keywords:** adolescent post-tubercular kyphotic, bone allograft, surgical treatment

## Abstract

This study aimed to investigate the mid-term outcome of ring-shaped bone allografts in the surgical treatment of adolescent post-tubercular kyphosis secondary to spinal tuberculosis.

The records of adolescent patients diagnosed with spinal tuberculosis who received treatment in our department between 2009 and 2013 were retrospectively reviewed. The anterior approach was used in cases of cervical kyphosis and the posterior approach was used in cases of thoracic and lumbar kyphosis. During the surgery, the ring-shaped bone was used as a structural bone graft associated with the cancellous bone filing in the center portion of the ring shape. Cobb's angle, signs of spinal infusion on computed tomography, and complications were followed up.

A total of 25 patients were included in our study. Among them, 3 involved the cervical region, 5 involved the thoracic region, 8 involved the thoracolumbar region, and 9 involved the lumbar region. The preoperative kyphosis deformity was a mean 65° Cobb's angle (40°–97°) compared to the postoperative 14° Cobb's angle (10°–21°) for an average correction of 51°. All wounds healed well without graft rejection. All patients achieved bone fusion 3 months postoperative for a 100% fusion rate.

Our results show that the ring-shaped allograft bone is an effective option for the treatment of adolescent kyphosis. The ring-shaped allograft bone demonstrated satisfactory mechanical strength and vertebral fusion without mid-term metallic toxicity.

## 1 Introduction

Spinal tuberculosis is a disease caused by *Mycobacterium tuberculosis* infection, which often leads to destruction of the involved vertebrae and progressive deformity of the vertebral alignment. In less developed countries or areas, spinal tuberculosis is the main cause of kyphosis deformity in adolescents because of the low-level health care system, with an estimated incidence of 0.5% to 8%.^[1,2]^ In Southwest China in particular, vast numbers of adolescent patients develop serious kyphosis secondary to spinal tuberculosis due to the lack of health services and early intervention. Unlike the pathological process of spinal tuberculosis in adults, adolescents often suffer from more severe and extensive vertebral destruction due to a sufficient blood supply around the epiphysis.^[3]^ Therefore, in these adolescents, the kyphosis deformity secondary to the spinal tuberculosis manifests aggressively and extensively, consequently resulting in severely impaired growth and quality of life.

Surgical treatment is presently the primary option for these patients. The standard surgical protocols include vertebrectomy and debridement, nerve decompression, and reconstruction of the spinal alignment with artificial internal implants.^[3,4]^ With respect to surgical issues, reconstruction of the normal vertebral alignment is most challenging. The surgical reconstruction must provide sufficient and lasting mechanical strength and spinal stability. The method of spine fusion, which fuses the anterior column rather than the middle and posterior columns, through internal fixation is considered the gold standard for surgical treatment. With the development of internal fixation, the use of a titanium cage or artificial vertebrae has proven encouraging in adults.^[5–9]^

However, challenges differ in the case of adolescents. First, more severely wedged vertebrae and kyphosis is common in adolescent patients because spinal tuberculosis often invades the anterior column of the spine and stops its growth while the other 2 columns continue growing and developing. Second, sufficient blood communication between vertebrae via the blood vessels of the epiphysis is prone to aggravating spinal destruction. Thus, tuberculosis could spread throughout the blood vessels in the epiphysis and lead to additional spinal segment involvement, which makes the treatment challenging. Third, adolescents are actively growing; therefore, metal toxicity of the implants is greater in adolescents than in adults.

Therefore, the spinal implants used to treat adolescent kyphosis should not only provide sustained and reliable mechanical strength for anterior column spinal fusion, such as the titanium cage or artificial vertebrae, but also avoid the potential metallic toxicity in the body preserving period. Thus, the metallic implants, which are the first choice in adults, are not suitable for adolescent patients. Similarly, despite being nontoxic and nononcogenic, autologous bone grafting is also not practical because the iliac and fibula, which are the main sources of autologous bone grafts, cannot meet the quantity demand during multisegment spinal reconstruction surgery. Additionally, the shapes of the iliac and fibula do not match those of the vertebrae and could cause significant problems during surgery.^[7,10]^

To avoid the aforementioned limitations, we have utilized allograft bone, which can be prefabricated into a shape matching the vertebrae as an alternative to the surgical treatment of adolescent kyphosis, for several years. The aim of the present study was to investigate the efficacy of the ring-shaped allograft for anterior column reconstruction for the treatment of kyphosis in adolescents after spinal tuberculosis.

## 2 Patients and methods

The cases of adolescent patients diagnosed with spinal tuberculosis who received surgical treatment in our department between 2009 and 2013 were retrospectively reviewed. Their demographic and medical information was collected from their medical files.

Spinal tuberculosis was confirmed by erythrocyte sedimentation rate, C-reactive protein, imaging findings, and intraoperative pathological examination.^[11]^ The surgical indications included apparent kyphosis deformity and nerve dysfunction. All patients took antituberculous drugs for 6 weeks before the surgery.

The anterior approach was used in cases of cervical kyphosis, while the posterior approach was used in cases of thoracic and lumbar kyphosis (Table 1). In the first step, the screw-rod system was used for the thoracic or lumbar spine, while the plate-screw system was used for the cervical spine. In the second step, debridement was performed. The deformed and wedged vertebrae and the intervertebral discs were removed (vertebrectomy or corpectomy). The spinal canal was decompressed if necessary. The third step was critical spinal reconstruction. The spinal alignment was regained via internal fixation, and the prefabricated and ring-shaped allograft bone with cancellous bone pellets filling in its center hollow portion was implanted to replace the removed vertebrae.

**Table 1:**
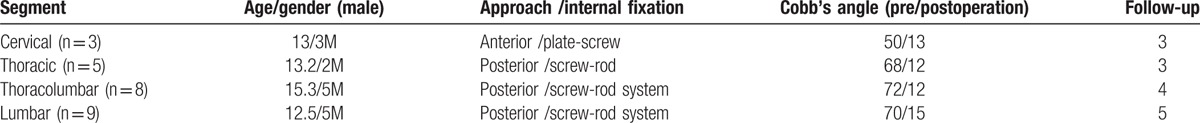
Preoperative information and postoperative follow-up.

A follow-up was performed every month since the operation. The follow-up measurements included Cobb's angle, sign of spinal infusion on computed tomography (CT), and complications.

All data were collected in blinded fashion by an attending doctor who was not involved in the study. The study was approved by the bioethics committee of Daping Hospital and all included patients provided informed formal consent.

## 3 Results

**Figure 1:**
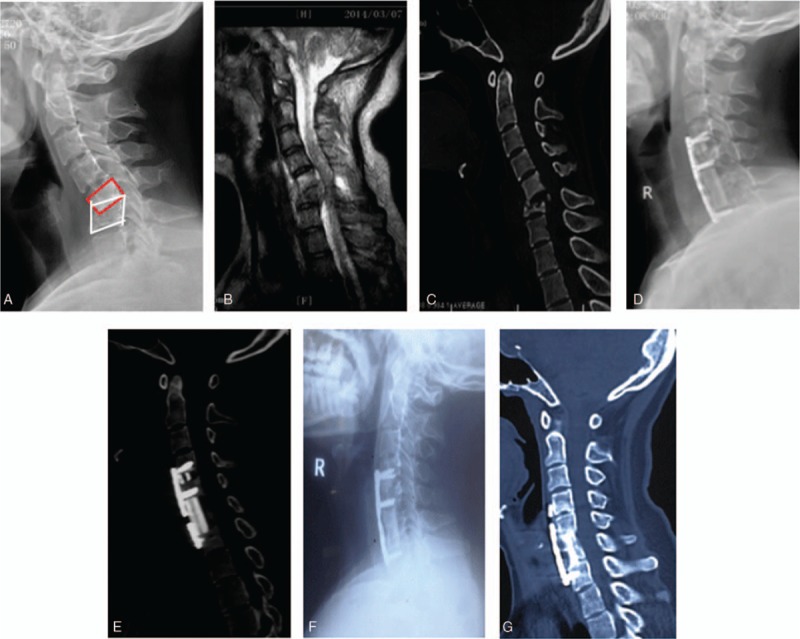
Cervical segment involvement. (A–C) Destruction of the C6 vertebral body, kyphosis deformity, and spinal canal encroachment; (D,E) spinal canal decompression, correction of kyphosis, and allograft after the operation; (F,G) bone fusion and well-maintained correction 1 year after the operation.

**Figure 2:**
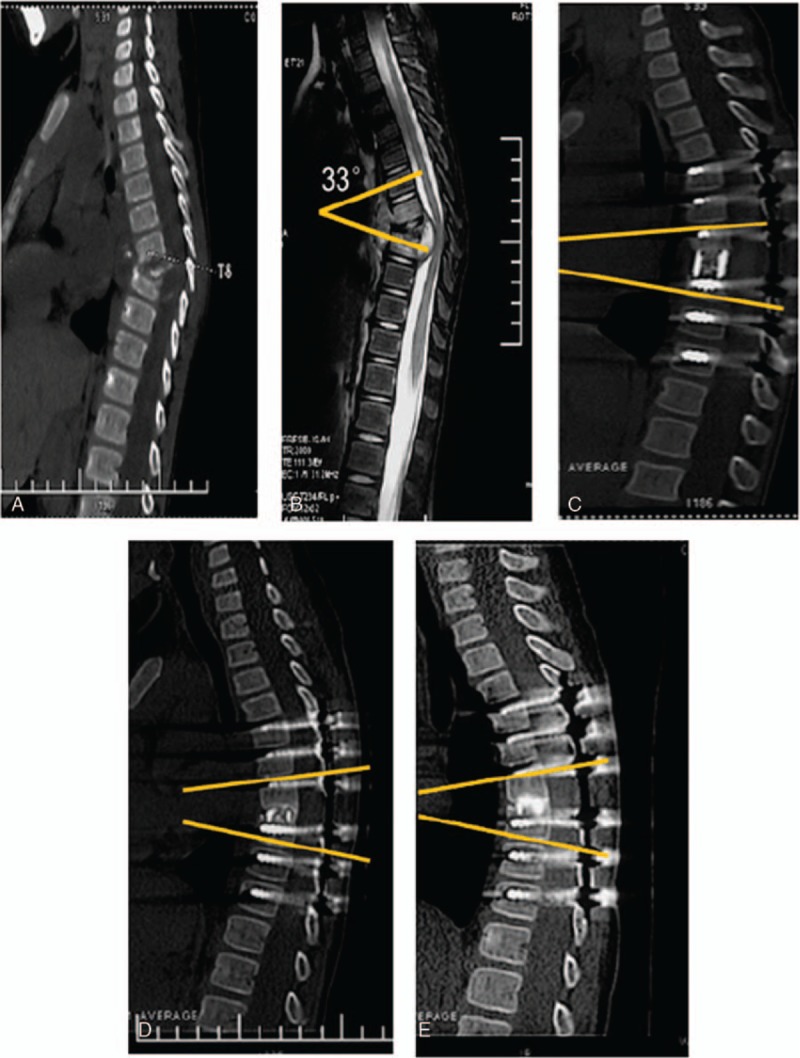
Thoracic segment involvement. (A,B) T9 vertebral destruction, kyphosis (Cobb's angle, 33°), and spinal canal compression; (C) after the operation (Cobb's angle, 12°); (D) 12 months after the operation (Cobb's angle, 16°); and (E) 30 months after the operation (Cobb's angle, 13°).

**Figure 3:**
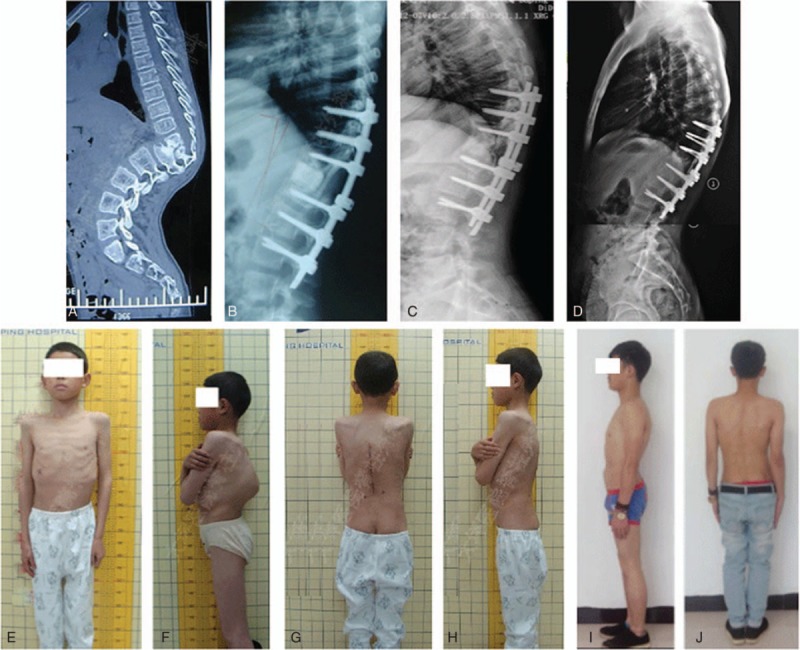
Thoracolumbar segment involvement. (A) Destruction of T11, T12, and L1 with a Cobb's angle of 91°; (B) after the operation; (C) 18 months after the operation; (D) 5 years after the operation (8° loss of correction); (E,F) appearance pre- and postoperative; (G) appearance 5 years after the operation without significant deformity but with an 8° loss of correction (proximal junctional kyphosis).

A total of 25 patients (15 males and 10 females) were included in our study. The mean patient age was 13.5 years (range, 5–18 years). Among these patients, the cervical region was involved in 3 (Fig. 1), thoracic in 5 (Fig. 2), thoracolumbar (T10-L1) in 8 (Fig. 3), and lumbar in 9. The average interval between diagnosis and operation was 1 week; 3 patients suffered the numbness and radicular pain around the thorax and weakness of both lower limbs; a 5-year-old child received surgery 3 days after the diagnosis because of severe neural symptoms. All patients were followed up for 3 years (range, 2–5 years). All patients experienced numbness. Two patients with cervical involvement also experienced radicular pain. No cases of dystaxia or cauda equina syndrome were found. At the last follow-up, the neural signs disappeared in all patients.

In this study, the preoperative kyphosis deformity was a mean 65°of Cobb's angle (range, 40°–97°), which decreased to a mean of 14° (range, 10°–21°) after the surgery for a mean correction of 51°. The corrections of the cervical, thoracic, thoracolumbar, and lumbar areas are shown in Table 1.

All wounds healed well without graft rejection. The numbness had disappeared in all patients at the last follow-up. All patients (100%) achieved bone fusion 3 months postoperatively according to the CT scan findings. Although 1 patient developed growth-induced junctional kyphosis (<10°), the spinal appearance was not affected.

## 4 Discussion

Therefore, the main objective of spinal surgery is to regain normal spinal morphology and stability.^[12]^ The titanium cage or artificial vertebrae associated with autogenous particulate bone grafting has been successfully used to treat adult spinal tuberculosis.^[13–15]^ Zhang et al^[18]^ reported a single-stage surgical protocol including debridement spinal reconstruction. Depending on the mechanical strength of the titanium cage or vertebrae, the reconstructed spinal structure can be maintained for a certain period. The bone fusion between the adjacent vertebrae provides long-term spinal stability.^[16–18]^ Papadopoulos et al's^[14]^ study showed that bone fusion failure was one of the most common complications of surgical treatment that often resulted in revision operations. Vamvanij et al^[15]^ stated that bone fusion was important for long-term effect. Compared with these previous studies, our results revealed the satisfactory long-term outcome without revision operations, as shown in Section 3.

When it comes to the potential metallic toxicity, our method avoided the artificial metal implants except for the internal fixation (actually the internal fixations were mostly removed finally due to the Chinese culture). The treatment strategy of adolescent spinal tuberculosis originates from that for adults, and similar surgical strategies have been widely used in clinical practice.^[14,18,19]^ However, the pathological characteristics of adolescent spinal tuberculosis differ from those of adult spinal tuberculosis, which is prone to breaking through the vertebral endplates and invading adjacent segments.^[3]^ Moreover, adolescents are actively growing; therefore, tissue compatibility and potential metallic toxicity of the metallic implants should be considered during treatment.^[7]^ Therefore, the titanium cage or artificial vertebrae, both of which are favored by adult patients, should not be considered the best choice. Nonmetallic or nonartificial bone grafts should be the first option for adolescents.^[10]^

Compared with the autogenous bone being widely accepted as the standard of bone grafts, its use is not suitable for adolescent spinal reconstruction. The iliac bone mainly provides cancellous bone and good osteogenesis, but it cannot offer sufficient mechanical strength. The limited quantities and donor complications of the iliac bones are additional challenges. The fibula has a long and thin shape that does not match that of the vertebrae, so it cannot offer sufficient mechanical strength. To solve this problem, Mosheiff et al^[20]^ and Govender et al^[19]^ proposed the use of a vascularized rib pedicle graft for tuberculosis kyphosis and presented a good fusion rate. However, the donor site morbidity after bone harvest and aesthetic issues are unfavorable.

Compared with the autogenesis bone graft, the ring-shaped bone allograft graft shows apparent advantages. First, the allograft bone ensures adequate bone sources. Second, the large ring-shaped bone can provide sufficient mechanical strength for spinal stability. Third, the ring-shaped bone has a similar shape and dimension to that of the vertebral body. Its cortical portion provided mechanical strength and structural stability. The hollow portion in its center can be filled with cancellous bone to provide reliable osteogenic activity. Finally, the bone is not an artificial implant and has no metallic toxicity.

As shown in the results, the kyphosis correction was well maintained during the follow-up period. The fusion rate was 100%, which favored the ring-shaped allograft as an effective treatment alternative for serious adolescent kyphosis. Although an 8° loss of correction and proximal junctional kyphosis recurred in 1 case, there was no obvious abnormal appearance or functional loss. The retrospective design and limited sample size are the main drawbacks of our study; however, the satisfactory results still indicate that the ring-shaped allograft is a safe and effective approach to treating adolescent spinal kyphosis following spinal tuberculosis.

## 5 Conclusion

The ring-shaped allograft bone without metallic toxicity can provide satisfactory mechanical strength and spinal fusion in the surgical treatment of adolescent kyphosis, while the ring-shaped allograft bone provides an effective treatment alternative.
